# Adherence to Mediterranean Diet and Diet Quality in Patients with Inflammatory Bowel Disease: A Single-Center, Observational, Case-Control Study

**DOI:** 10.3390/nu16111557

**Published:** 2024-05-21

**Authors:** Marta Cadoni, Agnese Favale, Rita Piras, Mauro Demurtas, Paola Soddu, Alessandra Usai, Ivan Ibba, Massimo Claudio Fantini, Sara Onali

**Affiliations:** 1 Department of Medical Science and Public Health, University of Cagliari, 09124 Cagliari, Italy; cdn.marta@gmail.com (M.C.); agnese.favale@unica.it (A.F.); soddupaola@gmail.com (P.S.); alessandra.usai@outlook.it (A.U.); sara.onali@unica.it (S.O.); 2 Azienda Ospedaliero-Universitaria di Cagliari, 09123 Cagliari, Italy; demurtasmau@gmail.com (M.D.); ibbaivan75@gmail.com (I.I.)

**Keywords:** Mediterranean diet, inflammatory bowel disease, ulcerative colitis, Crohn’s disease, diet quality, Medi-Lite score, DQI-I

## Abstract

The nutritional status in inflammatory bowel disease (IBD) is often impaired, and adherence to the Mediterranean diet (MedDiet) remains under-investigated. The aim of this study was to assess diet quality (DQ) and adherence to MedDiet in a cohort of Sardinian IBD patients. We conducted a case-control study in which 50 Crohn’s disease (CD) and 50 ulcerative colitis (UC) patients were matched with 100 healthy controls each. The Diet Quality Index (DQI-I) and Medi-Lite were used to assess DQ and adherence to MedDiet, respectively. Subgroup analysis by disease characteristics and use of advanced therapies were also carried out. DQI-I scored significantly lower in IBD, independently of disease localization and behavior (CD) and disease extent (UC): [DQI-I: CD 34.5 (IQR 33–37) vs. CTRL 40 (IQR 38.5–43) *p* < 0.0001; UC 34.5 (IQR 33–37) vs. CTRL 42 (IQR 40–44) *p* < 0.0001]. Medi-Lite scores were significantly lower in stricturing and ileo-colonic CD and in extensive UC: [Medi-Lite CD 7.5 (IQR 7–9)] vs. CTRL 9 (IQR 7–10) *p* = 0.0379]; [UC 8 (IQR7–10) vs. CTRL 9 (IQR 8–10.5) *p* = 0.0046]. IBD patients had a low DQ independently of disease type and phenotype. Patients with ileo-colonic stenosing CD or extensive UC had lower MedDiet adherence, suggesting that its benefits may be mitigated by low acceptance in specific subgroups.

## 1. Introduction

The chronic inflammatory process of the gut observed in ulcerative colitis (UC) and Crohn’s disease (CD), the major forms of inflammatory bowel disease (IBD), is responsible for structural damage and functional impairment of the gut. The nutritional status of patients affected by IBD is often impaired [1]. Malnutrition can occur both in UC and CD patients, although it is a considerably greater problem in the latter, given the capacity of CD to affect any part of the gastrointestinal tract (GI), potentially affecting the absorptive function of the gut. Additionally, the disease generally leads to lifestyle modification and changes in patients’ eating habits. Indeed, dietary changes might help reduce symptoms such as abdominal pain and diarrhea [2]. Patients with IBD also have an increased risk of self-imposed dietary restrictions, often based on personal or wrong beliefs, that may lead to the avoidance of certain foods or food groups in order to manage disease-related symptoms, with significant negative consequences on patients’ health [2,3]. Since IBD patients are at high risk for malnutrition, they should be screened and managed accordingly [4].

Although, to date, no diet has been proven to promote remission during active IBD, and none has been shown to control symptoms, both during disease flare-ups (enteral nutrition, parenteral nutrition, and Crohn’s disease exclusion diet) and during remission (specific carbohydrate diet (SCD), gluten-free diet, low-FODMAP diet, or autoimmune diet), different dietary regimens for IBD are under investigation. Among them, the Mediterranean diet (MedDiet) has received considerable attention.

The MedDiet mainly consists of a high intake of extra virgin olive oil and vegetables (including leafy green vegetables, fruits, cereals, nuts, and pulses/legumes), a moderate intake of fish, meat, dairy products, and red wine, and a low intake of eggs and sweets [5]. This dietary pattern is rich in antioxidant vitamins (vitamin C, vitamin E, β-carotene), minerals, natural folates, and phytochemicals (flavonoids), and it has demonstrated an significant role in reducing inflammation and in hindering the progression of chronic diseases [4]. Although there are limited data on the impact of a MedDiet on the course of IBD, adherence to a MedDiet has already been demonstrated to be beneficial in other chronic inflammatory diseases such as rheumatoid arthritis, where it significantly improved patients’ global assessment [6], as well as psoriasis, where a MedDiet has been associated with a reduction of psoriasis severity scores [7].

The fiber-rich MedDiet also has positive effects on the microbiota composition, particularly by increasing the percentage of fiber-degrading bacteria and the production of short-chain fatty acids, which are believed to play a positive effect in intestinal epithelial barrier function and to have a preventive effect in IBD [4,8]. Additionally, adherence to a short-term MedDiet leads to an improvement in anthropometric variables related to the development of metabolic syndrome, a reduction in liver steatosis, and in disease activity indexes in both CD and UC patients [9]. Finally, a MedDiet holds long-term health benefits such as a reduction of cardiovascular diseases and cancer [10]. Nevertheless, IBD patients have been shown to have low adherence to the MedDiet [2], though these studies were conducted in geographical areas where the MedDiet might not be diffused in the general population and where access to the MedDiet is limited.

That is why the aim of this study was to assess the adherence to a MedDiet and diet quality in an IBD population from a geographical area with a high prevalence of MedDiet compared to the general population. To this end, we analyzed MedDiet adherence and quality of diet in a Sardinian IBD population matched to healthy controls from the same region with respect to age, gender, and body mass index (BMI).

## 2. Materials and Methods

### 2.1. Study Population and Data Collection

We conducted a single-center, observational, case-control study. Patients affected by UC and CD were consecutively recruited at the IBD Unit of the University Hospital Monserrato, University of Cagliari, after providing written informed consent for privacy. UC and CD patients were matched 1:2 with healthy volunteers according to age, gender, and BMI. Clinical, demographic, lifestyle, and disease-related characteristics were collected at baseline from our electronic clinical records or through a direct interview during outpatients’ visits and included in a common database. Inclusion criteria required participants to be 18 years of age or older and able to understand and sign the written informed consent. UC or CD patients must have had a diagnosis for more than 6 months, must have been in remission or with mild disease activity according to the Harwey–Bradshaw Index (HBI ≤ 7) for CD and by partial Mayo score (pMayo ≤ 4) for UC. UC and CD patients with a history of total colectomy with oostomy or ileo-pouch anastomosis were excluded.

Adherence to the MedDiet was studied through the Medi-Lite questionnaire, in which nine food categories were considered: (1) fruit; (2) vegetables; (3) cereal grains; (4) legumes; (5) fish and fish products; (6) meat and meat products; (7) dairy products; (8) alcohol intake; and (9) olive oil. For food groups typical of the MedDiet (fruit, vegetables, cereals, legumes, and fish), a value of 2 was assigned to the highest category of consumption, 1 for the middle category, and 0 for the lowest category. Conversely, for food groups not typical of the MedDiet (meat and meat products, dairy products), a value of 2 was assigned for the lowest category, 1 for the middle category, and 0 for the highest category of consumption. For alcohol, the categories related to the alcohol unit (1 alcohol unit = 12 g of pure alcohol) were used, by assigning 2 points to the middle category (1–2 alcohol units/d), 1 point to the lowest category (1 alcohol unit/d), and 0 points to the highest category of consumption. Finally, 2 points were assigned for regular use of olive oil, 1 point for frequent use, and 0 points for occasional use. The final score was obtained by summing these values, and it varied from 0 (low adherence) to 18 (high adherence) [11]. Quality of diet was assessed with the Diet Quality Index (DQI-I) that emphasizes four major aspects of a high-quality, healthy diet: variety, adequacy, moderation, and overall balance [12]. Subgroup analysis of disease considering phenotypic characteristics, the use of advanced therapies (biologics or small molecules), and need for surgery was conducted.

### 2.2. Outcomes

The primary outcome of the study was to compare MedDiet adherence and the general diet quality in UC and CD patients to a reference population with similar demographic characteristics. Secondary outcomes included the comparative analysis of MedDiet adherence and quality of diet among patients with different disease phenotypes (i.e., location and behavior for CD and disease extent for UC), need for advanced therapies (biologics or small molecules such as JAK inhibitors), need for surgery, and the identification of patient- and disease-related factors independently correlated with MedDiet adherence and quality of diet.

### 2.3. Statistical Analysis

Differences between groups were analyzed by descriptive statistics indicating median and interquartile range or mean and standard deviation for continuous variables and number and percent of the total for categorical variables. For continuous and discrete variables, Wilcoxon nonparametric test and Fisher exact test, respectively, were used to assess statistical significance of the observed difference. A difference was considered significant if *p*-value < 0.05. Correlation between patient- and disease-related variables and MedDiet adherence and quality of diet was analyzed by univariate and multivariate linear regression. STATA 18.0 software (StataCorp, College Station, TX, USA) was used for statistical analysis. Since differences in adherence to MedDiet or quality of diet in IBD patients were never compared to that of a reference population, it was not possible to properly estimate the sample size. Nevertheless, we considered 50 patients per disease type (i.e., UC and CD) matched 1:2 with healthy control individuals acceptable for a pilot evaluation.

The research project was approved by the Ethics Board (Prot. PG/2018/15554). The study was conducted according to the Helsinki Declaration.

## 3. Results

### 3.1. IBD Study Population and Matching

Between February 2022 and March 2023, 50 CD and an equal number of UC patients in clinical remission were included and matched 1:2 with control subjects considering age, gender, and BMI as matching variables. The demographic and relevant clinical characteristics of IBD patients and their matched controls are reported in Table 1. The median age of CD patients was 43.5 (IQR 31–60) years and for UC patients was 47.5 (IQR 32–56) years. Median BMI was 22.8 (IQR 20.5–26.1) and 23.1 (IQR 20.5–26.1) for CD and UC, respectively. The female sex represented 46% of CD and 56% of UC patients. As expected, age, gender proportion, and BMI were not statistically different in the control groups. In contrast, regular physical activity and smoking habits were more frequent among controls of both CD and UC, while no difference was observed for diabetes and metabolic syndrome.

The median disease duration was 10 (IQR 5–13) and 9 (IQR 5–18) years for CD and UC, respectively (Table 2). Among CD patients, 29/50 (58%), 14/50 (28%), and 7/50 (14%) had a B1, B2, and B3 disease phenotype, respectively, according to the Montreal classification, and two thirds of patients [30/50 (60%)] had ileo-colonic localization of disease. A total of 22 (44%) CD patients suffered from perianal disease. Most UC patients [34/50 (68%)] had extensive colitis. In total, 33 CD patients (66%) vs. 22/50 (44%) UC patients were receiving advanced therapies, while almost all CD and UC patients were in clinical remission by HBI and partial Mayo score [CD 48/50 (96%); UC 47/50 (94%)], respectively. A total of 12 CD patients underwent previous CD-related surgery (24%).

### 3.2. Adherence to MedDiet and Quality of Diet in CD and UC Patients

**Figure 1:**
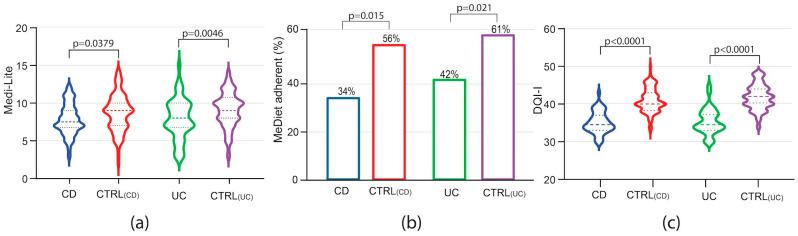
Medi-Lite score (**a**), adherence to the MedDiet (**b**) and DQI-I (**c**) in patients affected by Crohn’s disease (CD), ulcerative colitis (UC) and their matched controls, CTRL_(CD)_ and CTRL_(UC)_, respectively. In violin plots, broken lines (---) indicate median and dotted lines (…) indicate interquartile range. Numbers above the bars indicate the percent of adherent patients.

Assessing adherence to a MedDiet among IBD patients, Medi-Lite scores were significantly lower in both CD and UC patients as compared to controls [Medi-Lite CD 7.5 (IQR 7–9) vs. CTRL 9 (IQR 7–10) *p* = 0.0379; Medi-Lite UC 8 (IQR7–10) vs. CTRL 9 (IQR 8–10.5) *p* = 0.0046, Figure 1a]. Considering 8.5 as the established cut-off point for adherence to the MedDiet [11], 34% (95% CI 20.4–47.6) and 42% (95% CI 27.8–56.2) of CD and UC patients, respectively, adhered to the MedDiet (MedDiet score > 8.4). This was significantly less than their control groups, where 56% (95% CI 46.1–65.9; *p* = 0.015) and 61% (95% CI 51.3–70.7; *p* = 0.021) among CTRL_CD_ and CTRL_UC_, respectively, were considered adherent (Figure 1b).

Interestingly, diet quality was also shown to be poorer among IBD patients, with respect to healthy controls, as measured by the DQI-I [DQI-I CD 34.5 (IQR 33–37) vs. CTRL_CD_ 40 (IQR 38.5–43) *p* < 0.0001; DQI-I UC 34.5 (IQR 33–37) vs. CTRL_UC_ 42 (IQR 40–44) *p* < 0.0001, Figure 1c). All these data indicate that not only adherence to the MedDiet but also diet quality was compromised in IBD patients as compared to non-IBD patients from the same geographical area with a high prevalence of the MedDiet.

### 3.3. Impact of IBD Phenotype on MedDiet Adherence and Quality of Diet

**Figure 2:**
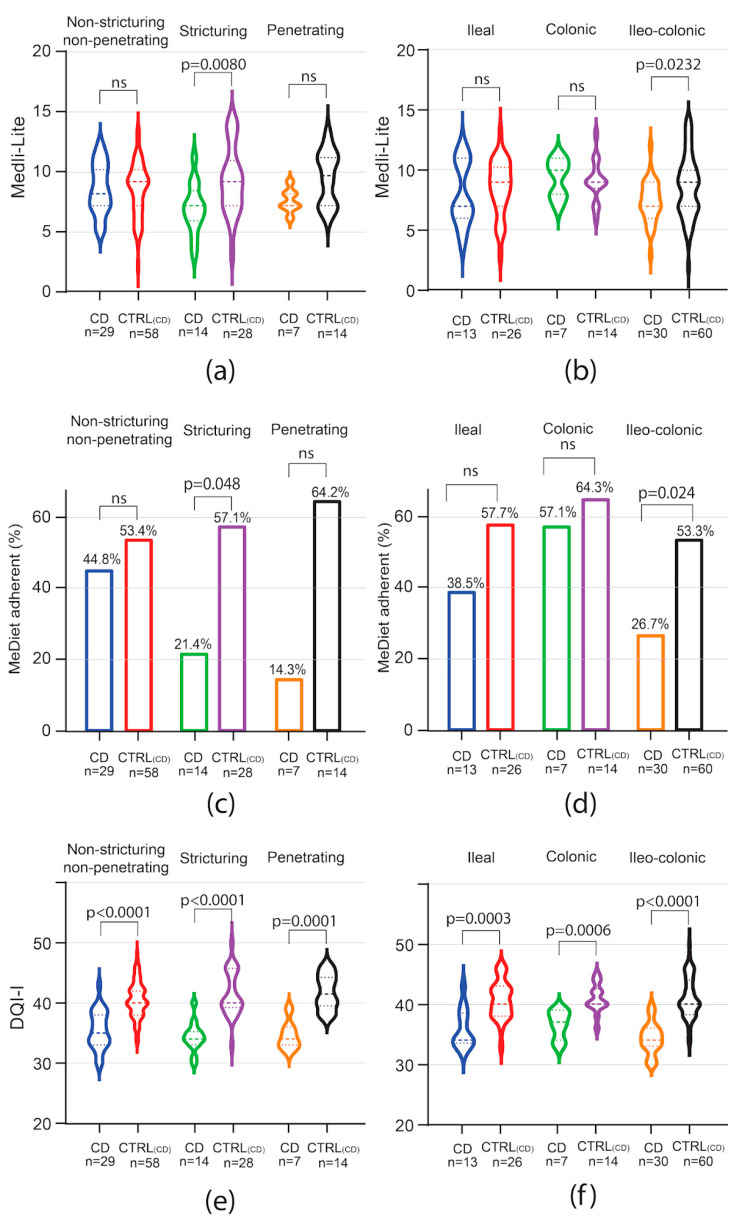
Impact of CD phenotype on Medi-Lite score, MedDiet adherence, and diet quality. Medi-Lite score (**a**,**b**), MedDiet adherence (**c**,**d**), and diet quality (**e**,**f**) by disease behavior (**a**,**c**,**e**) and phenotype (**b**,**d**,**f**). In violin plots, broken lines (---) indicate median and dotted lines (…) indicate interquartile range. Numbers above the bars indicate the percent of adherent patients.

In the CD subgroup analysis, Medi-Lite score was significantly lower in patients with stricturing phenotype [CD 7 (IQR 6–8) vs. 9 CTRL_CD_ (IQR 7–10.5) *p* = 0.0080] and ileo-colonic localization [CD 7 (IQR 6–9) vs. 9 CTRL_CD_ (IQR 7–10) *p* = 0.0232 Figure 2a,b]. Accordingly, adherence to the MedDiet was significantly lower in patients with stricturing disease [CD 21.4% (95% CI 3.15–46.0) vs. 57.1% CTRL_CD_ (95% CI 37.6–76.7), *p* = 0.048, Figure 2c]. Patients affected by penetrating disease also showed lower adherence as compared to controls, but the difference did not reach statistical significance [CD 14.3% (95% CI 20.6–49.2) vs. CTRL_CD_ 64.2% (95% CI 35.6–93.0), *p* = 0.063, Figure 2c]. Adherence to the MedDiet was also affected by disease location in CD patients. Indeed, while adherence to the MedDiet did not differ among patients affected by colonic disease, lower adherence was observed among patients affected by ileal or ileo-colonic disease, although the difference was statistically significant only in the latter [CD 26.7% (95% CI 9.87–43.5) vs. CTRL_CD_ 53.3% (95% CI 40.3–66.3), *p* = 0.024, Figure 2d].

**Figure 3:**
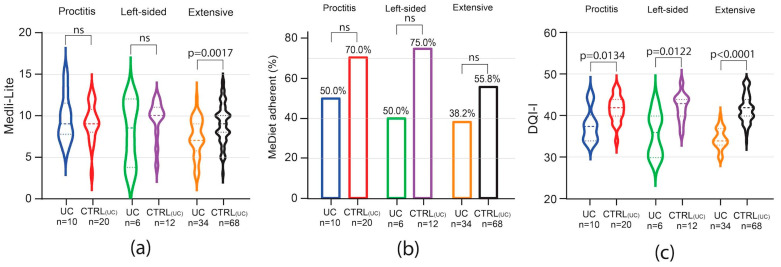
Impact of UC phenotype on Medi-Lite score, MedDiet adherence, and diet quality. Medi-Lite score (**a**), MedDiet adherence (**b**), and diet quality (**c**) by disease extent. In violin plots, broken lines (---) indicate median and dotted lines (…) indicate interquartile range. Numbers above the bars indicate the percent of adherent patients.

Among UC patients, Medi-Lite score was significantly lower only in those with extensive colitis [UC 7 (IQR 6–9) vs. 9 CTRL_UC_ (IQR 8–10) *p* = 0.0017], but resulted comparable to that of matched controls in patients affected by proctitis and left-sided colitis (Figure 3a). A trend of low adherence to a MedDiet was observed in all subgroups, but in none was this trend statistically significant (Figure 3b).

In contrast to MedDiet adherence, the results in overall diet quality as scored by the DQI-I index were negatively affected independently of the disease phenotype in both CD and UC. Indeed, among CD patients, DQI-I score was significantly lower independently of disease phenotype and location, with the higher difference observed in the subgroup of patients affected by stenosing and ileo-colonic disease (Figure 2e,f). In UC patients, independently of disease extent, DQI-I scored significantly lower than matched controls, with the highest difference observed in patients affected by extensive colitis (Figure 3c).

### 3.4. Impact of Need for Advanced Therapy and Surgery on MedDiet Adherence and Quality

**Figure 4:**
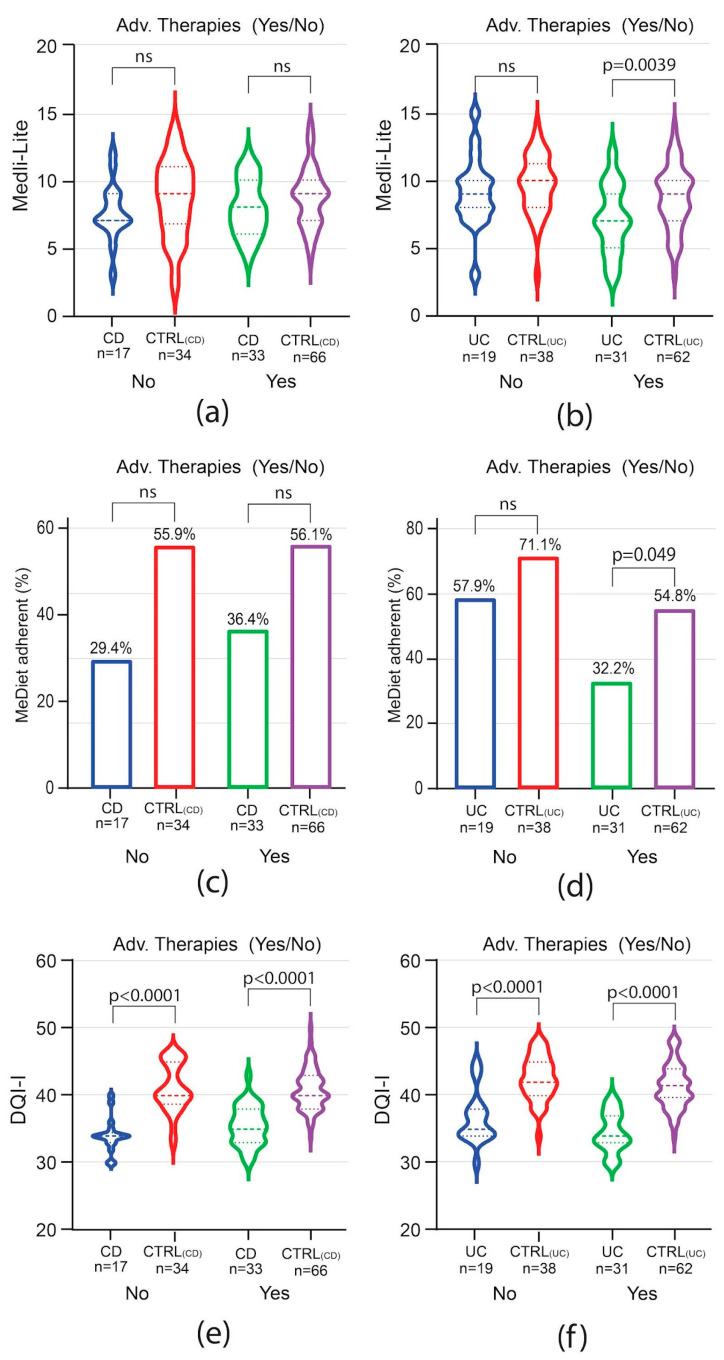
Impact of need for advanced therapy in IBD on MedDiet adherence and quality. Medi-Lite score (**a**,**b**), MedDiet adherence (**c**,**d**), and diet quality (**e**,**f**) in CD vs. CTRL_(CD)_ (**a**,**c**,**e**) and in UC vs. CTRL_(UC)_ (**b**,**d**,**f**). In violin plots, broken lines (---) indicate median and dotted lines (…) indicate interquartile range. Numbers above the bars indicate the percent of adherent patients.

**Figure 5:**
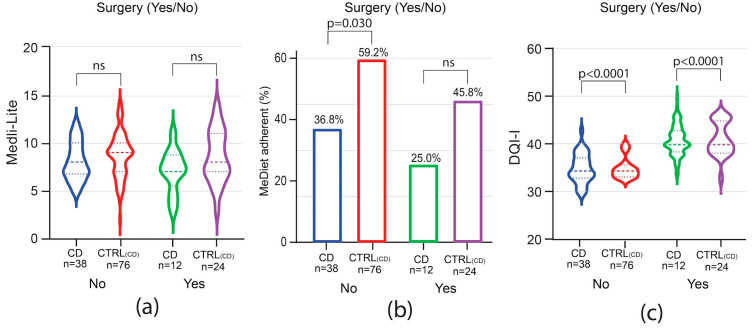
Impact of surgery on Medi-Lite score, MedDiet adherence, and DQI-I. Medi-Lite score (**a**), MedDiet adherence (**b**), and diet quality (**c**). In violin plots, broken lines (---) indicate median and dotted lines (…) indicate interquartile range. Numbers above the bars indicate the percent of adherent patients.

To evaluate whether the disease course impacted adherence to the MedDiet and the overall diet quality, patients were analyzed by groups based on the need for advanced therapies and, for CD only, on previous bowel surgery. Among CD patients, the need for advanced therapies did not influence Medi-Lite score as compared to matched controls (Figure 4a). A trend of lower adherence to the MedDiet was observed among CD patients independently of the need for advanced therapies, but it was not statistically significant (Figure 4c). In contrast to CD patients, in UC patients, Medi-Lite score and adherence to the MedDiet were significantly lower among those needing advanced therapies [Medi-Lite UC 7 (IQR 5–9) vs. 9 CTRL_UC_ (IQR 7–10) *p* = 0.0039; MedDiet adherent UC 32.2% (95% CI 14.8–49.7) vs. CTRL_UC_ 54.8% (95% CI 42.1–67.6), *p* = 0.049, Figure 4b–d]. DQI-I scores were significantly lower in both CD and UC no matter which therapy was adopted (Figure 4e,f). We also explored previous surgery as a potential factor influencing the dietary habit of CD patients. Medi-Lite score did not significantly differ from matched controls independently of a history of bowel surgery (Figure 5a). However, the number of patients adherent to a MedDiet was lower in CD patients as compared to control, though statistical significance was observed only in patients without previous surgery (Figure 5b). Similarly, DQI-I was significantly reduced in CD patients, with surgery having no influence (Figure 5c).

### 3.5. Univariate and Multivariate Correlation Analysis

To assess whether Medi-Lite and DQI-I correlated with clinical and phenotypic disease variables, univariate and multivariate linear regression analysis was conducted. In CD patients, the univariate analysis of Medi-Lite scores showed significant correlation with stricturing disease behavior (Coeff. −1.436, *p* = 0.027) and ileal or ileocolonic location (Coeff. −1.704, *p* = 0.043, Table 3). However, when modeling Medi-Lite correlation using both location and behavior, none of the scores were found to be significantly correlated. In the univariate analysis, only an age at diagnosis >40 years inversely correlated with DQI-I score (Coeff. −1.895, *p* = 0.031) in CD patients. In UC patients, by contrst, Medi-Lite score positively correlated with age at diagnosis ≤40 (Coeff. 1.667, *p* = 0.038), while it was inversely correlated with extended colitis (Coeff. −1.617, *p* = 0.048) and the need for advanced therapies (Coeff. −2.028, *p* = 0.009). However, only the need for advanced therapies remained significantly correlated when considering all three variables in the same regression model (advanced therapies Coeff. −1.568, *p* = 0.048). A negative correlation between DQI-I and disease extent (Coeff. −2.412, *p* = 0.019) and the need for advanced therapies (Coeff. −2.136, *p* = 0.032) was observed in the univariate analysis in UC patients, but the effect was lost when multivariate correlation analysis was performed. Medi-Lite adherence was not associated with any of the variables considered in the analysis of both UC and CD patients.

## 4. Discussion

The main aim of this study was to assess the adherence to a MedDiet and the general deity quality in a cohort of IBD patients and control individuals, matched by age, sex, and BMI, living in a geographical area with a high prevalence of Mediterranean diet. To this end, we analyzed the data from a population of IBD patients and matched controls living in Sardinia, an island located in the heart of the Mediterranean Sea. To assess adherence to the MedDiet and general diet quality, we used two validated questionnaires. The Medi-Lite is a quantitative score based on the literature and elaborated on the basis of data derived from cohort studies evaluating the relationship between MedDiet adherence and health outcomes. At the same time, the general diet quality of single individuals constituting our study cohorts was assessed by DQI-I, an index created to compare the quality of diets from different geographical areas which takes in consideration variety, adequacy, moderation, and overall balance. Both adherence to MedDiet and general diet quality were shown to be significantly lower in CD and UC patients compared to those observed in control individuals. These data are in line with the well-known observation that modification of the daily diet in IBD patients is a common event. Indeed, up to 68% of these patients self-impose dietary restrictions, primarily due to the personal belief that food has a major impact on triggering symptoms and disease flares [13,14]. This behavior may, in turn, dampen their QoL; indeed, up to 66% of patients declared that they deprived themselves of their favorite foods, and roughly 20% of them renounced dining out [13].

In an Italian cohort of CD and UC patients, Fiorindi et al. reported that CD patients with inactive disease scored higher in their adherence to a MedDiet, while no difference was observed in UC patients [5]. Similarly, in a Greek cohort of CD patients, adherence to a MedDiet was lower in patients with active disease as compared to those in remission [15]. In our cohort of IBD patients, deviation of MedDiet adherence and diet quality from matched controls was not driven by disease activity. Indeed, about 95% of patients in our IBD cohort were in clinical remission, with less than 5% showing mild disease activity. Our data are in line with those that reported that while active disease in CD patients was associated with higher prevalence of low protein intake and lower intakes of carbohydrates, fibers, fruits, vegetables, legumes, and sweets, as well as lower adherence to the MedDiet compared to those in remission, CD patients still had low adherence to the MedDiet and showed inadequate diet quality while in clinical remission [16]. These data indicate that the deviation from an adequate MedDiet and low diet quality might be independent of symptom relief during disease flares, but it could persist during the phase of disease remission, probably due to a physiologic carryover effect leading to a persistent modification of nutritional habits. Of note, these dietary restrictions and the consequent reduced diet quality may cause or worsen the well-known IBD-related malnutrition, which has been extensively associated with poor IBD outcomes, including increased number and duration of hospitalizations, higher mortality, increased number of emergency department visits, more frequent non-elective CD surgeries, as well as reduced response to medical therapy [10,17,18,19].

In our cohort, disease phenotype influenced adherence to the MedDiet. Among CD patients, stricturing behavior and ileal involvement correlated with lower MedDiet adherence, while the same was observed among patients with extensive colitis among UC patients. In stricturing CD patients, especially in those patients where the terminal ileum was involved, patients are more susceptible to sub-occlusive symptoms; self-imposed or healthcare practitioner-suggested limitation in fiber and fruit consumption might prevent acute obstructive episodes and relieve abdominal pain. Extensive colitis might potentially be characterized by more severe symptoms and disease course. In these settings, a more restricted food intake and the avoidance of specific aliments could justify the divergence from the MedDiet in these patients. Although a low-fiber diet is reasonable for patients with active symptomatic IBD, dietary management, in the long-term, should have the goal of reintroducing soluble fibers, fresh fruits, and vegetables. Indeed, a prospective, randomized study has suggested that both the MedDiet and SCD were equally effective in achieving clinical remission and reduced calprotectin levels [20].

Also noteworthy is the finding that diet quality was independent of disease behavior, though disease behavior was higher in those subgroups where low adherence to the MedDiet was observed (i.e., those with ileal stricturing disease among CD patients and extensive colitis among UC patients). This observation suggests that the disease has a major impact on diet quality, while avoidance of specific aliments, responsible for the divergence from the MedDiet, is more dependent on the phenotype of the disease.

Considering the need for advanced therapies, we found that results in MediLite score and adherence to MedDiet were significantly lower among UC but not CD patients as compared to their matched controls. In CD, a trend of lower adherence to the MedDiet, though not statistically significant, was observed independently of advanced therapy. Similar results were obtained stratifying CD patients by previous surgery, with no difference in Medi-Lite score and a trend towards lower adherence to the MedDiet (statistical significance was observed only in patients without surgery) independently of surgical history. While these results suggest that the need for advanced therapy in UC patients might indicate a more aggressive disease and more severe symptoms with a higher impact on dietary habits, in CD patients, these results should be interpreted cautiously. Indeed, in these patients, the use of advanced therapies might not reflect the severity of disease symptoms. The disconnection between disease severity and the severity of symptoms in CD patients might explain why patients with relatively mild symptoms can be aggressively treated with advanced therapies in an attempt to slow disease progression and to prevent disease-related complications. Accordingly, a history of bowel surgical resection, which can be considered a proxy of severe disease course but not disease symptoms severity, did not significantly impact MedDiet adherence or have any effect on diet quality reduction observed among CD patients. These data are also supported by univariate and multivariate correlation analysis performed in UC and CD groups of patients. In UC patients, adherence to the Medi-Lite score correlated with age at diagnosis, disease extent, and need for advanced therapy in univariate analysis, with the need for advanced therapy remaining the only significant variable when analyzed together. In contrast, the need for advanced therapies did not correlate with Medi-Lite score, further supporting the different impacts this diagnosis may have on patients’ diet and the different ramifications of these impacts for UC and CD patients in terms of nutritional habits.

Our study highlights that quality of diet is impaired in both UC and CD patients independently of their disease localization, phenotype, need for advanced therapy, or previous surgery (the latter applies only for CD), confirming the altered dietary habits of IBD patients. Food avoidance should be discouraged in IBD patients, bearing in mind that they carry a high risk of malnutrition and nutritional deficiencies.

Accordingly, adherence to a healthy MedDiet should be highly encouraged, as should consideration for the potential benefit of reducing the intake of ultra-processed foods, which have been implicated in the worsening of intestinal inflammation in animal models of colitis. Indeed, in interleukin-10-deficient (IL10KO) mice which develop spontaneous colitis, food additive-specific alterations in the microbiome and host–microbe interactions accelerated disease onset [21]. Moreover, EDTA salts have been shown to induce intestinal inflammation and to increase colorectal carcinogenesis in an IBD model of colitis-associated colorectal cancer at doses comparable to those commonly observed in humans; this occurs through the disruption of the physiologic epithelial barrier function [22]. Interestingly, food additive use has been associated with increased emergency department admissions, number of hospitalizations, and odds of having elevated inflammatory biomarkers in IBD patients [23,24,25,26].

Recently, a prospective cohort study showed that IBD patients with a healthy lifestyle, who were defined as having a good adherence to the MedDiet, and who performed physical activity had lower risk of moderate and severe relapses [adjusted Hazard ratio (aHR), 0.250; 95% CI, 0.093–0.670] and steroid use (aHR 0.292; 95% CI, 0.103–0.828) [27].

Consequently, screening for good diet quality and offering correct dietary interventions and instructions should be primary goals in IBD settings.

Limitations of our study include the lack of objective measures of nutritional parameters. These were beyond the goal of the study, which aimed to describe adherence to the MedDiet and diet quality, not nutritional status, which was, nevertheless, alluded to through the assessment of BMI, although this is a parameter with known flaws. Another limitation is the use of advanced therapies as a proxy for severe disease course. Indeed, as discussed, patients with CD are often treated with advanced therapies earlier in the course of their disease and independently of disease severity. However, we believe that this parameter could have provided an objective measure of disease severity in the absence of other validated clinical parameters for IBD patients in clinical remission.

We also believe this study had some points of strength. To our knowledge, this is the first case-control study, realized in a typical Mediterranean geographic area, and aiming to measure adherence to the MedDiet, calculated by a validated tool, and the quality of diet in both CD and UC patients in clinical remission compared to matched healthy controls. While similar studies explored diet quality or adherence to the MedDiet while limiting analysis to IBD patients, in this study, we compared CD and UC patients with matched controls. This allowed us to measure the extent of IBD’s effect on MedDiet adherence and diet quality using a local reference population instead of data coming from the general population.

## 5. Conclusions

In conclusion, in our study, IBD was associated with an overall low quality of diet independently of disease type and phenotype. However, Medi-Lite detected a lower adherence to Mediterranean diet in patients affected by ileocolonic stenosing CD disease and in UC patients with extensive colitis, suggesting that the potential benefit of the Mediterranean diet may be mitigated by low acceptance in specific IBD patient subgroups.

## Figures and Tables

**Table 1:** Description of demographic and relevant clinical data of CD and UC patients and their matched controls.

		CD (*n* = 50)	CTRL_(CD)_ (*n* = 100)	*p*	UC (*n* = 50)	CTRL_(UC)_ (*n* = 100)	*p*
**Age (yrs)**	Median (IQR)	43.5 (31–60)	42.5 (32–57)	0.646	47.5 (32–56)	47 (29.5–57)	0.971
**Gender (F)**	*n* (%)	23 (46)	46 (46)	1	28 (56)	56 (46)	1
**BMI**	Median (IQR)	22.8 (20.5–26.1)	23.8 (21.3–26.3)	0.477	23.1 (20.5–26.1)	22.75 (20.3–25.8)	0.592
**Active smoking**	*n* (%)	1 (2)	31 (31)	<0.001	0	25 (25)	<0.001
**Physical Activity**	*n* (%)	0	44 (44)	<0.001	2 (4)	44 (44)	<0.001
**Diabetes**	*n* (%)	4 (8)	3 (3)	0.362	1 (2)	0	0.333
**Metabolic syndrome**	*n* (%)	1 (2)	3 (3)	1	0	1 (1)	1

BMI, body mass index; CD, Crohn’s disease; CTRL, healthy controls; UC, ulcerative colitis. Data are presented as number (*n*) and percentage (%) for categorical variables and as median (interquartile range) for continuous variables. *p*-values represent statistical significance between groups.

**Table 2:** Description of clinical variables of CD and UC patients.

	CD (*n* = 50)	UC (*n* = 50)
**Disease duration (yrs)** median (IQR)	10 (5–13)	9 (5–18)
**Age (yrs) at diagnosis *n* (%)**		
A1 ≤ 16	13 (10)	5 (6)
A2 17–40	30 (60)	30 (60)
A3 > 40	15 (30)	17 (34)
**Disease behavior *n* (%)**		
B1 non-stricturing non-penetrating	29 (58)	--
B2 stricturing	14 (28)	--
B3 penetrating	7 (14)	--
**Disease localization *n* (%)**		
L1 Ileal	13 (26)	--
L2 Colonic	7 (14)	--
L3 Ileo-colonic	39 (60)	--
L4 Proximal loc.	0	--
**Perianal disease***n* (%)	22 (44)	
**Disease extent**		
E1 proctitis *n* (%)	--	10 (20)
E2 Left-sided *n* (%)	--	6 (12)
E3 Extensive *n* (%)	--	34 (68)
**HBI** mean (±SD)	1.04 (±1.44)	--
**Mayo score** mean (±SD)	--	0.52 (±0.89)
**Clinical Remission** *n* (%)	48 (96)	47 (94)
**Previous bowel surgery** *n* (%)	12 (24)	
**Advanced therapies** *n* (%)	33 (66)	31 (62)
**Immunosuppressant**	5 (10)	15 (30)

CD, Crohn’s disease; UC, ulcerative colitis; HBI, Harwey–Bradshaw Index. Data are presented as number (*n*) and percentage (%) for categorical variables and as mean ± standard deviation or median (interquartile range) for continuous variables.

**Table 3:** Univariate and multivariate correlation analysis of CD and UC patients.

CD		Univariate				Multivariate			
Medi-Lite		Coeff.	95% CI		*p*	Coeff.	95% CI		*p*
Age at diagnosis	>40	−0.981	−2.251	0.289	0.127				
Behavior	Stenosing	−1.436	−2.6098	−0.175	0.027	−1.18	−2.464	0.104	0.071
Location	Ileal/ileo-colonic	−1.704	−3.351	−0.057	0.043	−1.32	−2.981	0.341	0.117
Adv Therapies	Yes	0.351	−0.904	1.607	0.576				
Gender	Male	0.172	−1.023	1.368	0.773				
Disease duration		−0.022	−0.099	0.053	0.546				
Surgery	Yes	−0.969	−2.338	0.399	0.161				
**DQI-I**		**Coeff.**	**95% CI**		***p***				
Age at diagnosis	>40	−1.895	−3.611	−0.179	0.031				
Behavior	Stenosing	−0.976	−2.793	0.84	0.285				
Location	Ileal/ileo-colonic	−1.757	−4.082	0.567	0.135				
Adv Therapies	yes	1.428	−0.265	3.121	0.096				
Gender	Male	0.613	−1.0334	2.261	0.458				
Disease duration		0.013	−0.091	0.119	0.793				
Surgery	yes	0.031	−1.903	1.964	0.975				
**MedDiet adherence**		**OR**	**95% CI**		***p***				
Age at diagnosis	>40	0.375	0.089	1.574	0.239				
Behavior	Stenosing	0.428	0.101	1.812	0.249				
Location	Ileal/ileo-colonic	0.325	0.063	1.662	0.177				
Adv Therapies	Yes	1.371	0.388	4.842	0.624				
Gender	Male	1.526	0.471	4.950	0.481				
Disease duration		0.953	0.876	1.038	0.271				
Surgery	yes	0.571	0.132	2.469	0.454				
**UC**		**Univariate**				**Multivariate**			
**Medi-Lite**		**Coeff.**	**95% CI**		***p***	**Coeff.**	**95% CI**		***p***
Age at diagnosis	>40	1.667	0.944	3.238	0.038	1.332	−0.18	2.843	0.083
Extent	Extended	−1.617	−3.228	−0.144	0.048	−0.861	−2.483	0.76	0.29
Adv Therapies	Yes	−2.028	−3.522	−0.535	0.009	−1.568	−3.125	−0.011	0.048
Gender	Male	−0.422	−1.987	1.142	0.059				
Disease duration		−0.025	−0.113	0.063	0.577				
**DQI-I**		**Coeff.**	**95% CI**		***p***	**Coeff.**	**95% CI**		***p***
Age at diagnosis	>40	1.326	−0.727	3.38	0.2				
Extent	Extended	−2.412	−4.415	−0.41	0.019	−1.866	−3.974	0.243	0.082
Adv Therapies	Yes	−2.136	−4.078	−0.193	0.032	−1.515	−3.541	0.511	0.139
Gender	Male	−1.062	−3.031	0.908	0.284				
Disease duration		−0.061	−0.172	0.051	0.279				
**MedDiet adherence**		**OR**	**95% CI**		***p***				
Age at diagnosis	>40	1.333	0.491	3.62	0.572				
Extent	Extended	0.619	0.186	2.054	0.433				
Adv Therapies	Yes	0.346	0.106	1.129	0.079				
Gender	Male	0.555	0.178	1.734	0.312				
Disease duration		0.97	0.907	1.037	0.377				

CD, Crohn’s disease; UC, ulcerative colitis; CI, confidence interval; OR, odds ratio. *p*-value considered statistically significant if <0.05.

## Data Availability

The original contributions presented in the study are included in the article, further inquiries can be directed to the corresponding author.
